# Supplementary material for: Targeting of the Fun30 nucleosome remodeller by the Dpb11 scaffold facilitates cell cycle-regulated DNA end resection
Source: eLife. 2017 Jan 12;6:e21687. doi: 10.7554/eLife.21687 (PMC5300703; doi:10.7554/eLife.21687)
Supplement: Supplementary file 1. — (A) Table 1 lists all S.cerevisiae yeast strains used in this study, their relevant genotypes and the source. (B) Table 2. Plasmids used in this study. Table 2 lists all yeast plasmids and mammalian expression vectors used in this study and their relevant features. DOI: http://dx.doi.org/10.7554/eLife.21687.031 [file elife-21687-supp1.docx]

**Supplementary file 1**

Table 1. Yeast strains used in this study.

| Strain | Relevant genotype | Source |
| --- | --- | --- |
| W303a | MATa ade2-1 ura3-1 his3-11,15 trp1-1 leu2-3,112 can1-100 | (Thomas and Rothstein, 1989) |
| pJ69-7a | MATa trp1-∆901 leu2-3,112 901 ura3-52 his3-∆200 gal4∆ gal8∆ GAL2-ADE2 LYS2::GAL1-HIS3 met2::GAL7-lacZ | (James et al, 1996) |
| YSB117 | MATa lys1∆::natNT2 pep4∆::LEU2 bar1∆::TRP1 | this study |
| YSB220 | MATa lys1∆::natNT2 pep4∆::LEU2 bar1∆::TRP1 Fun30-3FLAG::hphNT1 | this study |
| YBP388 | MATa pep4∆::LEU2 | this study |
| YSB760 | MATa pep4∆::LEU2 Fun30-3FLAG::hphNT1 | this study |
| YSB707 | MATa cdc28-as1 bar1∆::trp1 pep4∆::leu2 Fun30-3FLAG | this study |
| YSB708 | MATa cdc28-as1 bar1∆::trp1 pep4∆::leu2 | this study |
| YSB714 | MATa bar1∆::TRP1 pep4∆::LEU2 natNT2::Fun30-S20A-3FLAG::hphNT1 | this study |
| YSB718 | MATa bar1∆::TRP1 pep4∆::LEU2 natNT2::Fun30-S28A-3FLAG::hphNT1 | this study |
| YSB719 | MATa bar1∆::TRP1 pep4∆::LEU2 natNT2::Fun30-SS20,28AA-3FLAG::hphNT1 | this study |
| YSB723 | MATa bar1∆::TRP1 pep4∆::LEU2 natNT2:: Fun30-SS20,28AA-dpb11∆N-3FLAG::hphNT1 | this study |
| YSB745 | MATa bar1∆::TRP1 pep4∆::LEU2 Fun30-3FLAG::hphNT1 | this study |
| YSB743 | MATa hml∆::prS hmr∆::pRS bar1∆::trp1 pGal-HO::ade3 Fun30-3FLAG::hphNT1 | this study |
| YSB725 | MATa hml∆::prS hmr∆::pRS bar1∆::trp1 pGal-HO::ade3 natNT2::Fun30-S20A-3FLAG::hphNT1 | this study |
| YSB727 | MATa hml∆::prS hmr∆::pRS bar1∆::trp1 pGal-HO::ade3 natNT2::Fun30-S28A-3FLAG::hphNT1 | this study |
| YSB728 | MATa hml∆::prS hmr∆::pRS bar1∆::trp1 pGal-HO::ade3 natNT2::Fun30-SS20,28AA-3FLAG::hphNT1 | this study |
| YSB731 | MATa hml∆::prS hmr∆::pRS bar1∆::trp1 pGal-HO::ade3 natNT2::Fun30-SS20,28AA-dpb11∆N-3FLAG::hphNT1 | this study |
| L40 | MATa his3∆200 trp1-901 leu2-3,112 ade2 LYS2::(4lexAop-HIS3) URA3::(8lexAop-lacZ) GAL4 | Invitrogen |
| YSB782 | MATa bar1∆::TRP1 pep4∆::LEU2 natNT2::Fun30-SS20,28AA-3FLAG::hphNT1 ddc1-9myc::kanMX4 | this study |
| YSB771 | MATa lys1∆::natNT2 pep4∆::LEU2 bar1∆::TRP1 ddc1-9myc::kanMX4 | this study |
| YSB772 | MATa lys1∆::natNT2 pep4∆::LEU2 bar1∆::TRP1 Fun30-3FLAG::hphNT1 ddc1-9myc::kanMX4 | this study |
| YSB753 | MATa bar1∆::TRP1 pep4∆::LEU2 Fun30-3FLAG::hphNT1 ddc1-T602A::kanMX4 | this study |
| YSB517 | MATa hml∆::prS hmr∆::pRS bar1∆::trp1 pGal-HO::ade3 | this study |
| YSB525 | MATa hml∆::prS hmr∆::pRS bar1∆::trp1 pGal-HO::ade3 fun30∆::hphNT1 | this study |
| YSB260 | YMV80 rad51∆::hphNT1 | this study |
| YSB329 | YMV80 fun30-S20A::TRP1 rad51∆::hphNT1 | this study |
| YSB330 | YMV80 fun30-S28A::TRP1 rad51∆::hphNT1 | this study |
| YSB331 | YMV80 fun30-SS20,28AA::TRP1 rad51∆::hphNT1 | this study |
| YSB253 | YMV80 fun30∆::kanMX4 rad51∆::hphNT1 | this study |
| UCC3511 | hmr::URA3 | (Singer et al, 1996) |
| YSB248 | hmr::URA3 fun30∆::hphNT1 | this study |
| YSB335 | hmr::URA3 fun30∆::hphNT1 Fun30 SS20,28AA::LEU2 | this study |
| AEY1017 | ChrVII-L-TEL::URA3 | (Meijsing et al, 2001) |
| YSB246 | ChrVII-L-TEL::URA3 fun30∆::hphNT1 | this study |
| YSB294 | ChrVII-L-TEL::URA3 fun30∆::hphNT1 Fun30::TRP1 | this study |
| YSB297 | ChrVII-L-TEL::URA3 fun30∆::hphNT1 Fun30 SS20,28AA::TRP1 | this study |
| YSB314 | MATa lys1∆::natNT2 pep4∆::LEU2 bar1∆::TRP1 fun30∆::hphNT1 | this study |
| YJW031 | MATa rad9∆::kanMX4 | this study |
| YDG148 | MATa fun30∆::hphNT1 | this study |
| YSB183 | MATa fun30∆::hphNT1 Fun30 SS20,28AA::TRP1 | this study |
| YJW032 | MATa fun30∆::hphNT1 rad9∆::kanMX4 | this study |
| YJW035 | MATa fun30∆::hphNT1 rad9∆::kanMX4 Fun30 SS20,28AA::TRP1 | this study |
| YSB758 | MATa fun30∆::kanMX4 | this study |
| YSB761 | MATa fun30∆::kanMX4 Ddc1-Fun30- SS20,28AA-3FLAG::hphNT1 | this study |
| YSB777 | MATa fun30∆::kanMX4 Ddc1-Fun30- SS20,28AA-K603R-3FLAG::hphNT1 | this study |
| YSB791 | MATa bar1∆::TRP1 pep4∆::LEU2 natNT2:: Fun30-SS20,28AA-K603R-dpb11∆N-3FLAG::hphNT1 | this study |
| YSB797 | MATa hml∆::prS hmr∆::pRS bar1∆::trp1 pGal-HO::ade3 fun30∆::kanMX4 Ddc1-Fun30- SS20,28AA-3FLAG::hphNT1 | this study |
| YSB819 | MATa fun30∆::kanMX4 pGAL-SMARCAD1 1-300-FUN30 30-C-3FLAG::URA3 | this study |
| YSB784 | MATa bar1∆::TRP1 pep4∆::LEU2 GAL4 pGAl1-10 Fun30-3FLAG::HIS3 | this study |
| YSB910 | MATa hml∆::prS hmr∆::pRS bar1∆::trp1 pGal-HO::ade3 Fun30-3FLAG::hphNT1 ura3::Sld3-dpb11∆N dpb11∆::kanMX4 | this study |
| YSB911 | MATa fun30∆::kanMX4 pGAL-GFP-FUN30 30-C-3FLAG::URA3 | this study |
| YSB832 | MATa hml∆::prS hmr∆::pRS bar1∆::trp1 pGal-HO::ade3 yku70∆::natNT2 | this study |

Table 2. Plasmids used in this study.

| name | description |
| --- | --- |
| pDG1 | pGAD-C1 Fun30 1-188 |
| pAD25 | pGAD-C1 Rad9 |
| pAD30 | pGAD-C1 Ddc1 |
| pBD23 | pGBD-C1 Dpb11 |
| pBD26 | pGBD-C1 Dpb11 1-276 |
| pUK211 | pB66 Dpb11 271-582 |
| pGAD-C1 | pGAD-C1 (James et al, 1996) |
| pGBD-C1 | pGBD-C1 (James et al, 1996) |
| pUK1 | pAG416 GPD-Dpb11 |
| pBP91 | pMALp2x RAD9 |
| pKR347 | pRS303 Fun30-3FLAG pGAL1-10 GAL4 (Fun30 ORF codon optimized for yeast expression) |
| pBP48 | pGex4T1 DPB11 1-275 |
| pSB035 | pGAD-C1 Fun30 1-188 S20A |
| pSB036 | pGAD-C1 Fun30 1-188 S28A |
| pSB029 | pGAD-C1 Fun30 1-188 SS20,28AA |
| pSB075 | pGAD-C1 Fun30 29-188 |
| pAP3 | pGAD-C1 Fun30 |
| pKS8 | pGAD-C1 Fun30 1-420 |
| pKS10 | pGAD-C1 Fun301-500 |
| pKS12 | pGAD-C1 Fun30 1-555 |
| pSB181 | pBTM116 Dpb11 1-276 |
| pSB31 | pRS304 Fun30 S20A |
| pSB32 | pRS304 Fun30 S28A |
| pSB33 | pRS304 Fun30 SS20,28AA |
| pSB140 | pRS304 Fun30 SS20,28AA-Dpb11 276-C |
| pSB38 | pRS305 Fun30 SS20,28AA |
| pDB104 | pB27 TOPBP1 1-360 |
| pSB190 | pB6 SMARCAD1 1-220 |
| pSB191 | pB6 SMARCAD1 1-220 T24A |
| pSB192 | pB6 SMARCAD1 1-220 S34A |
| pSB193 | pB6 SMARCAD1 1-220 T54A |
| pSB194 | pB6 SMARCAD1 1-220 T71A |
| pSB195 | pB6 SMARCAD1 1-220 T103A |
| pSB205 | pB6 SMARCAD1 55-274 |
| pSB174 | pB6 SMARCAD1 55-274 T71A |
| pSB189 | Yiplac211 pGAL-SMARCAD1 1-300-FUN30 30-C-3FLAG |
| pSB196 | Yiplac211 pGAL-FUN30 30-C-3FLAG |
| pSB247 | Yiplac211 pGAL-GFP-FUN30 30-C-3FLAG |
| pSB242 | Yiplac211 pSld3-oSld3-dpb11∆N |
| pSB206 | pB27 TOPBP1 1-766 |
| pSB197 | pB27 TOPBP1 1-360 K155E |
| pSB198 | pB27 TOPBP1 1-360 KK154,155AM |
| pSB199 | pB27 TOPBP1 1-360 K250E |
| pSB202 | pB27 TOPBP1 1-766 K155E |
| pSB203 | pB27 TOPBP1 1-766 KK154,155AM |
| pSB204 | pB27 TOPBP1 1-766 K250E |
| pPF345 | pCS2-GFP-SMARCAD1-55-445 |
| pPF348 | pCS2-GFP-SMARCAD1-55-274 |
| pPF351 | pCS2-GFP-SMARCAD1-55-274-T71A |
